# Supplementary figures and images for: Hyperelongate ornamental tail feathers in a new early Cretaceous enantiornithine bird
Source: PLoS One. 2026 May 27;21(5):e0347641. doi: 10.1371/journal.pone.0347641 (PMC13215521; doi:10.1371/journal.pone.0347641)

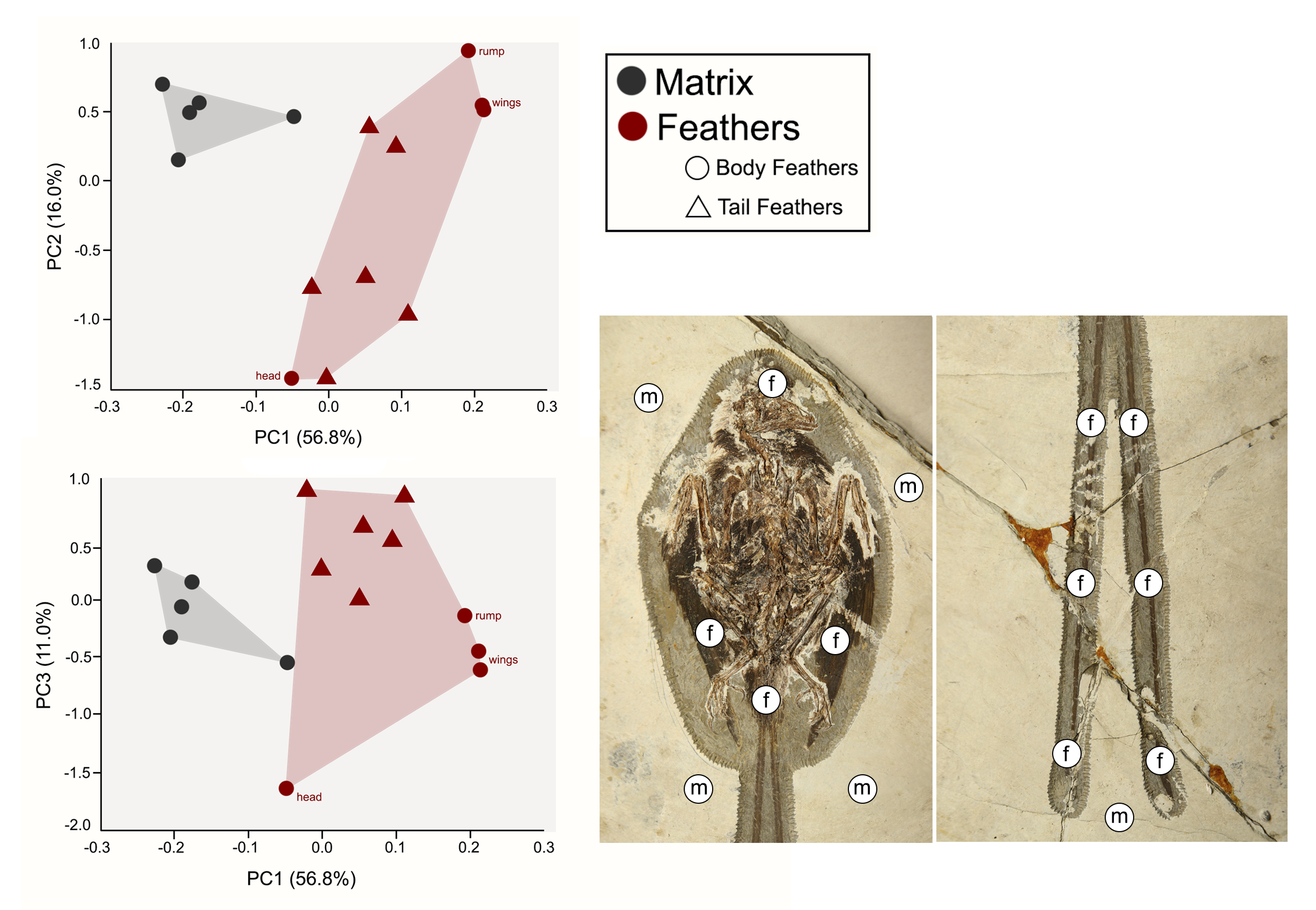

Supplement: S1 Fig — Feathers and matrix exhibit different chemical signatures, supporting the true presence of both the body covering and the elongate tail feathers. PC1–3 represent abundances of significant elements (detailed in Table S1). Inset photos of the specimen show where samples were taken. Abbreviations: f, feather; m, matrix. (PNG) [file pone.0347641.s003.png]

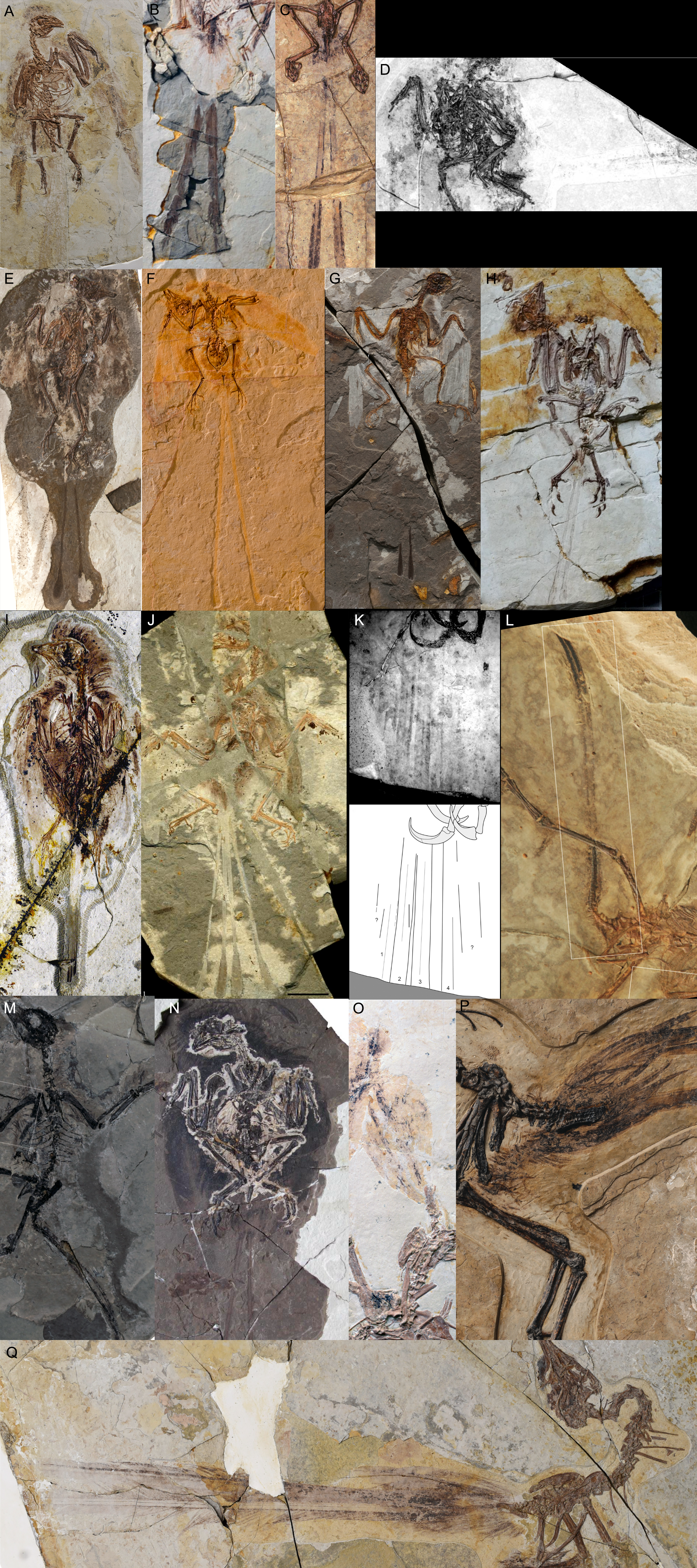

Supplement: S2 Fig — (A) Parapengornis [34], (B) Eopengornis [99], (C) indet. Enantiornithine GSGM-07-CM-001; Rackets – (D) Bohaiornis [12], (E) Dapingfangornis [5], (F) Junornis [58], (G) Orienantius [40], (H) Misuvavis [42], (I) Protopteryx [57], (J) Paraprotopteryx [50], (K) Shanweiniao [14], (L) Enantiornithine indet. IVPP V 15564 [55], (M) Enantiornithine indet. STM 34−9 [55], (N) Bohaiornithid indet. CUGB P1202 [23]; Fanned or multiple morphotypes – (O) Chiappeavis [6], (P) Feitianius [100] , (Q) Yuanchuavis [101] . (PNG) [file pone.0347641.s004.png]

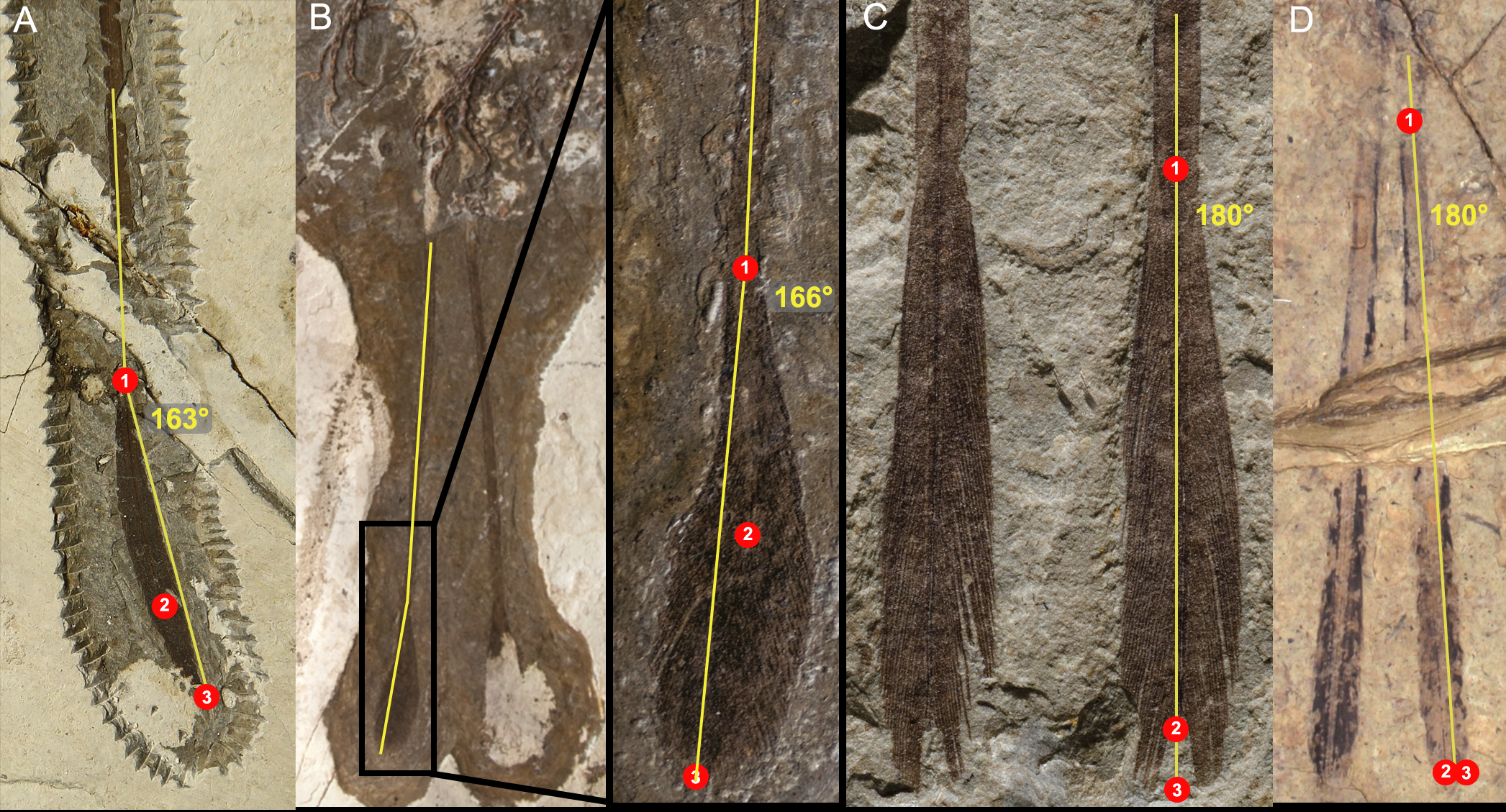

Supplement: S3 Fig — Proximally to distally, the red points indicate (1) the start of the racket, (2) the termination of the rachis, and (3) the distal margin of the racket. Yellow lines measure the angle using the pre-racket rachis along the midline, the start of the racket, and the racket’s distal central margin. Angles were measured using ImageJ. (A) Plumadraco, (B) Dapingfangornis (C) Orienantius, and (D) CAGS-IG-07-CM-001. Note the proportional differences between points 2 and 3 among sampled taxa with enfeeblement occurring within (A) and (B) more so than (C) and (D). A greater distance between these points would facilitate greater movement of the intermediate space, as made evident by angular differences among Plumadraco and Dapingfangornis compared to Orienantius and CAGS-IG-07-CM-001 (which show no discernable angular difference). (PNG) [file pone.0347641.s005.png]
