# Supplementary material for: Hyperelongate ornamental tail feathers in a new early Cretaceous enantiornithine bird
Source: PLoS One. 2026 May 27;21(5):e0347641. doi: 10.1371/journal.pone.0347641 (PMC13215521; doi:10.1371/journal.pone.0347641)
Supplement: Attachment — Submitted filename: Response to Reviewers.docx [file pone.0347641.s007.docx]

Editorial comments requiring responses

Please take this opportunity to be sure you have met all of our guidelines for new species. For proper registration of a new zoological taxon, we require two specific statements to be included in your manuscript.

We have included the required LSID values for the new taxon underneath the introduction of its new name

We note that Figure 6 and 7 in your submission contain copyrighted images. All PLOS content is published under the Creative Commons Attribution License (CC BY 4.0), which means that the manuscript, images, and Supporting Information files will be freely available online, and any third party is permitted to access, download, copy, distribute, and use these materials in any way, even commercially, with proper attribution. For more information, see our copyright guidelines: http://journals.plos.org/plosone/s/licenses-and-copyright.

Figure 6E is the only illustration created by someone who was not an author. Permissions have been secured and the document attached as directed.

Reviewer #1: General Assessment
Specific Recommendations for Clarification
Nesting Ecology and Morphology Correlations:
Clarification is needed regarding the inferences linking nesting ecology to tail morphology. Correlations should be treated with caution, as the specific ecology of this new taxon may differ from other fossils used for comparison. Please ensure that assumptions about nesting behavior do not overextend the available evidence.

Thank you very much for this suggestion. We have made sure to not overextend our interpretations drawn from other enantiornithines – only that, based on available evidence, our conclusions suggest a shared ground-nesting strategy across enantiornithines. We outline the available evidence from three different enantiornithines across three different geological times to suggest that this behavior is likely shared across the group – based on all available nesting evidence within this group. We then discuss the behavioral implications of this nesting strategy in modern birds and how this would affect enantiornithine behavior and probably parental roles (and likely in all birds across time). We have made sure to edit the text to also reflect the uncertainty that the reviewer has pointed out. Thank you!

Definition of Rachis-Dominated Feathers (RDFs):
The morphological definition of RDFs requires further illustration. Typically, "rachis-dominated" implies that the rachis comprises the major part of the feather structure; however, barbs are clearly observed in this specimen (particularly within the rackets). Please reconcile this terminology with the observed morphology to avoid confusion.

We have modified the definition of “RDF” in our introduction to state that they are “feathers with proportionately wide rachi throughout *majority of their lengths*, referred to as rachis-dominated feathers”. By stating “majority”, we eliminate confusion that they maintain their wide width for the entire feather – which tends to never happen in any enantiornithine RDF with the full length preserved.

Phylogenetic Support Values:
Please provide support indices (e.g., bootstrap or jackknife values) for the TNT phylogenetic analysis. Including these statistical values will strengthen the robustness of the cladistic results.

Given that are results reflect those of a 50% majority tree, we find these values to be unnecessary.

Pygostyle Morphology and Display Mechanics:
Fan-shaped tail feathers and rectrices are generally understood to have evolved in Neornithines, often associated with a short pygostyle. However, in Enantiornithines, which possess an elongate pygostyle, the mechanical effect of this structure on tail feather display remains unclear. Please discuss how the long, shaped pygostyle might influence tail feather display mechanics compared to neornithines.

Thank you for the suggestion, and a great point to bring up! The full biomechanical effects of elongate enantiornithine pygostyles on rectricial flexion is a topic that cannot be adequately covered in this manuscript as it entails another major physics-based manuscript-length report. For example, a longer moment arm (longer pygostyle) results in faster flexion and more exaggerated speed the further you move from the fulcrum – but these results and discussion require research outside the scope of this manuscript. These pygostyles would have undoubtedly affected the mechanical advantage and moment arm mechanics across taxa (e.g., pengornithids – broad and short, compared to bohaiornithids – distally tapered and long).

In trying to avoid overextending the evidence we have, we have only discussed the plausible implications of pygostyle morphology/insertion surfaces in the discussion (543-550).

Reviewer #2: **Comments to the Author(s)**

This study describes a new enantiornithine bird and reports an unusual hyperelongate ornamental tail feather among early birds. Overall, this manuscript is well-written and the points are quite straightforward. I only have a few suggestions and recommendations that I believe might improve the quality of this manuscript:

1) Lines 21–22: In the abstract, I am not sure whether this comparison is appropriate — passerines today are considerably more diverse, clearly far exceeding the diversity level seen in enantiornithines.

Revised. Mention of passerines has been removed

2) Line 115: It is not very common to place the measurement table immediately here. Consider moving it to the location where measurements are first mentioned in the text.
Revised. Now moved into the results section

3) Line 123: It would be more appropriate to refer to the holotype rather than simply the "fossil specimen" of Plumadraco.
Revised

4) Lines 145–154: In the Diagnosis section, it would be better to separate the characters that support referral to Enantiornithes from those that further support assignment to Bohaiornithidae. Additionally, many of the features used to assign this taxon to bohaiornithids — such as the furcula, coracoid, sternum, and sacral vertebrae — are indicated as crushed in the line drawing figure.
Revised. The features that associate this individual with bohaiornithids are now clarified. Portions of the furcula, coracoid, sternum, and sacral vertebrae, while crushed, allow for enough morphological description that indicate both enantiornithine and specifically, bohaiornithid affinities.

5) Line 153: Please indicate that this refers specifically to the lateral trabeculae of the sternum.
Revised

6) Lines 209–210: The rami in this new specimen are slightly bowed. Could this bowing account for the apparently narrow interclavicular angle, or might there be inconsistency in how this angle is measured across taxa?
The angle is measured from the distal-most omal tips to the midline of the joined rami. The lateral bowing margins do not affect the inter-clavicular angle.

7) Line 214: It is unclear whether "pterygoma" is the appropriate term for the coracoid body.
Revised. “pterygoma” changed to “corpus”

8) Lines 230–232: To me, the left trabecula appears more caudolaterally oriented rather than purely caudally oriented.
Agreed – thank you for this suggestion. This section has been revised.

9) Line 266: Since the ungual phalanx of the minor digit is commonly very tiny and easily lost during preservation, it may be better not to place too much emphasis on the phalangeal formula being 2-3-1 here.
Revised and removed

10) Lines 370–371: It is unclear how a precise 3D structure can be reconstructed from 2D observations alone.
Three dimensional enantiornithine RDFs are used as a template to infer the homologous structure also likely present in *Plumadraco*.

11) Line 402: This should read "exceptional preservation of RDFs".
Revised

12) Lines 406–407: In Figure 6D, it would be helpful to indicate the exact location of the barbs within the tail feather of the modern bird-of-paradise.
Thank you for the suggestion. But in the manuscript and in the figure caption, we state that they are “occipital” plumes. These feathers actually appear as-in throughout their length, erupting from just behind the eye.

13) Lines 426–429: The use of percentage values may be appropriate for Confuciusornis, given the large number of available specimens, but may not be applicable to enantiornithines, for which most taxa are represented by very few specimens.
We very much agree, which is why we only do so for the only, currently published, enantiornithine with multiple specimens preserving soft tissues (*Protopteryx*).

14) Lines 453–454: The authors discuss differences in RDF morphology between STM11-4 and other fossil birds, yet only Orienantius is included in the comparison shown in Figure 7. It would be helpful to include additional enantiornithine tail feathers in this figure for a more comprehensive comparison.

Thank you for the suggestion. Other enantiornithines bearing elongate RDFs with their distal tips present are figured in Fig. S3. The primary objective of figure 7 is the comparison with extant birds to illustrate that there are in fact differences in distal rachis termination which results in varying levels of plasticity/rigidity.
